# Biofunctionalisation of Polypyrrole Nanowires Array with Sulfite Oxidase Coupled with the Integration of Platinum Nanoparticles for Ultrasensitive Amperometric Detection of Sulfite

**DOI:** 10.3390/bios13060621

**Published:** 2023-06-05

**Authors:** Shahid Hussain, Samuel B. Adeloju

**Affiliations:** 1 School of Chemistry, Monash University, Clayton, VIC 3800, Australia; dr.sha@hotmail.com; 2 Faculty of Science & Health, Charles Sturt University, Albury, NSW 2640, Australia

**Keywords:** sulfite, sulfite oxidase, amperometric detection, AAO template, PPy nanowires, Pt nanoparticles

## Abstract

Sulfite determination in foods and alcoholic beverages is a common requirement by food and drug administration organisations in most countries. In this study, the enzyme, sulfite oxidase (SOx), is used to biofunctionalise a platinum-nanoparticle-modified polypyrrole nanowire array (PPyNWA) for the ultrasensitive amperometric detection of sulfite. A dual-step anodisation method was used to prepare the anodic aluminum oxide membrane used as a template for the initial fabrication of the PPyNWA. PtNPs were subsequently deposited on the PPyNWA by potential cycling in a platinum solution. The resulting PPyNWA-PtNP electrode was then biofuntionalised by adsorption of SOx onto the surface. The confirmation of the adsorption of SOx and the presence of PtNPs in the PPyNWA-PtNPs-SOx biosensor was verified by scanning electron microscopy and electron dispersive X-ray spectroscopy. Cyclic voltammetry and amperometric measurements were used to investigate the properties of the nanobiosensor and to optimise its use for sulfite detection. Ultrasensitive detection of sulfite with the PPyNWA-PtNPs-SOx nanobiosensor was accomplished by use of 0.3 M pyrrole, 10 U mL^−1^ of SOx, adsorption time of 8 h, a polymerisation period of 900 s, and an applied current density of 0.7 mA cm^−2^. The response time of the nanobiosensor was 2 s, and its excellent analytical performance was substantiated with a sensitivity of 57.33 μA cm^−2^ mM^−1^, a limit of detection of 12.35 nM, and a linear response range from 0.12 to 1200 μM. Application of the nanobiosensor to sulfite determination in beer and wine samples was achieved with a recovery efficiency of 97–103%.

## 1. Introduction

The synthesis of new biomaterials has attracted much interest in the past two to three decades, and these have gained increasing use for fabrication of various sensors, biosensors, clinical diagnostics, and implants [1]. Invariably, the achievement of the desired biomaterials often involves the use of various surface modification strategies [2,3]. In particular, biofunctionalisation has emerged as a surface modification strategy of choice that has attracted considerable interest and applications. This approach enables a desired biofunctionality to be introduced by immobilisation of biomolecules, such as antibodies, DNA, enzymes, peptides, polysaccharides, proteins, and RNA, on the chosen materials [4,5,6]. The use of enzymes, in particular, for biofunctionalisation has attracted much interest because of their unique advantages, which include the ease of handling, conferment of selectivity and specificity for desired analytes, reusability of the immobilised enzymes, reduced cost of the immobilised enzymes, reduced diffusion limitations, and improved catalytic activities [4].

In recent years, biofunctionalisation has gained considerable interest for the development of nanosensors and nanobiosensors because of the considerable improvement in the stability of biomolecules and their associated improvement in sensitivity, with greatly reduced concentrations of biomolecules. The use of enzymes, in particular, as biofunctional agents on nanomaterials often improves the stability of the immobilised enzyme, while also enabling repeated use [7,8]. This has, to date, formed the basis for the fabrication of numerous reported nanobiosensors. However, while carbon nanotubes, graphene, and metallic nanoparticles are the more commonly used nanomaterials for this purpose, biofunctionalisation of nanowire arrays (NWA) is still not as common. Yet, nanowires and NWA have the desired properties for improving stability and analytical performance due to their low weight and associated unique multifunctional and physicochemical properties [9]. Due to their large surface area and availability of conduction channels, nanowires used for sensors and biosensors are capable of increasing sensitivity, improving response time, and enabling the achievement of much lower detection limits [10]. All these factors are significantly important for improving the reliability of the existing biosensing methods for determination of various substances.

We have previously used glucose oxidase [11] and pyruvate oxidase [12,13] with a gel support to biofunctionalise NWA for the detection of glucose and phosphate. In those studies, we achieved excellent detection limits of 50 and 100 nM for glucose and phosphate, respectively. We also applied the resulting nanobiosensors for their detection in blood and water samples. Due to our ongoing interest in the detection of sulfite and its extensive use as a preservative in foods, pharmaceuticals, and beverages [14,15,16], as well as the wide-spread attention due to the toxic effects of sulfites on human health [17], the use of sulfite oxidase (SOx) is investigated in this study for the biofunctionalisation of NWA to achieve ultrasensitive detection of sulfite. To our knowledge, no previous biofunctionalisation of NWA with SOx has been reported. Furthermore, unlike in our previous studies, where bovine serum albumin mixed with glutaraldehyde was used to achieve biofunctionalisation of NWA, we explored a novel route in this study involving the formation of PPyNWA decorated with platinum nanoparticles (PtNPs) and subsequent biofunctionalisation with SOx by adsorption to achieve better stability and improved performance. For this purpose, we used an AAO template to fabricate the membrane upon which the PPy film is grown and, subsequently, biofunctionalised with SOx. We adopted this approach because template synthesis is one of the best approaches for producing well-aligned nanowires [18]. In particular, the fabrication of NWAs by using potentiostatic electrodeposition combined with anodic aluminum oxide (AAO) templates facilitates controlled growth by applied electrochemical parameters [19]. This method is suitably effective for the growth of nanowires with significant hardness, consistent pore size, and considerable pore density, which are amenable for low-cost and large-scale processing with the use of a relatively simple equipment [19]. We anticipate that the use of PtNPs to decorate the PPyNWA will help improve the surface morphology and, due to its biocompatibility, enable high biomolecule-loading capacity and large surface area, while further improving electron transport between the sensing biomolecules and the electrode. Furthermore, to optimise the resulting SOx-functionalised PPyNWA-PtNPs (as a PPyNWA-PtNPs-SOx biosensor), the influence of pyrrole concentration for PPyNWA, chosen current density, polymerisation period, SOx concentration, deposition of PtNPs, scan rate, buffer concentration, and pH was carefully investigated. In addition, we investigated the application and reliability of the PPyNWA-PtNPs-SOx biosensor for the determination of sulfite in some alcoholic beverages.

## 2. Materials and Methods

### 2.1. Reagents

Aluminum foil of high purity (99.99%) was supplied by Beijing Cuibolin Non-Ferrous Technology Developing Co., Ltd., Beijing, China. Chromic acid, oxalic acid, phosphoric acid, pyrrole, potassium chloride, potassium hexachloroplatinate (K_2_PtCl_6_), sodium sulfite, sulfuric acid, sulfite oxidase (EC 1.8.3.1, 32 unit mg^−1^ protein), and other chemicals were obtained from Sigma-Aldrich Company, Sydney, Australia. Prior to use, the distillation of pyrrole was carried out at 130 °C, and to prevent UV degradation and air oxidation, it was stored at −2 to −10 °C in a container covered with aluminum foil. Milli-Q water was used to prepare all solutions. Supporting electrolyte used for all measurements was 0.05 M phosphate buffer solution (pH 7.0). Before each analysis, fresh sulfite solutions were prepared and diluted as needed. Spectroquant sulfite test kit (Merck photometric method) was used to obtain comparative results for concentrations of sulfite present in some wine and beer samples.

### 2.2. Apparatus

An HP 6443B DC power supply was used to anodise AAO templates. A computer-connected potentiostat/galvanostat was used for electro-polymerisation and amperometric detection with a three-electrode cell, which consisted of a reference (Ag/AgCl/3 M KCl) electrode, an auxiliary (Pt wire) electrode, and a working (1 mm^2^ Pt disk) electrode. AAO template was fixed with Pt working electrode for fabrication of PPy nanowires and further studies. An EDAQ potentiostat (low-noise and high-sensitivity), which utilised the EChem™ data acquisition software and was connected to an EDAQ e-corder 401, was used for all voltammetric and impedance spectroscopic measurements. Milli-Q water was used to prepare all solutions. For comparison studies, sulfite was determined in wines and beer samples with spectroquant NOVA 60 (Merck Pty Ltd, Darmstadt, Germany). JEOL JSM-6300F (JEOL Australasi Pty Ltd, Frenchs Forest, NSW, Australia) scanning electron microscope was used to conduct all surface morphological studies.

### 2.3. Construction of Modified PPyNWA-PtNPs-SOx Electrode

**Scheme 1:**
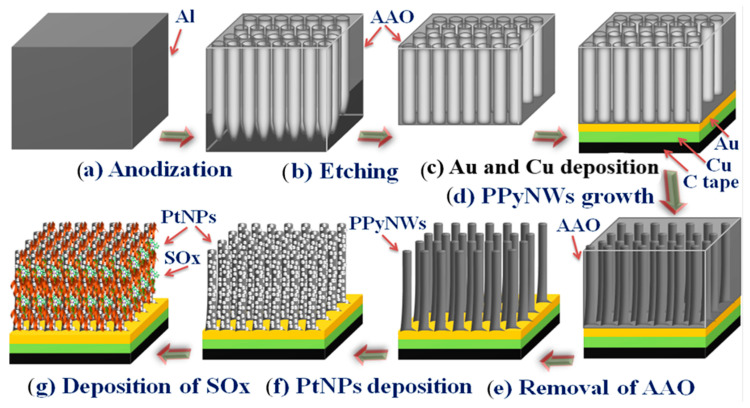
Sequential steps involved in the (**a**,**b**) formation of the AAO template from aluminum disc by double anodisation and removal of barrier layer, (**c**) gold coating, (**d**) formation of PPyNWA by electropolymerisation of pyrrole onto AAO template, (**e**) removal of AAO template, (**f**) deposition of PtNPs on PPyNWA, and (**g**) biofunctionalisation with SOx.

The sulfite biosensor was designed based on the modification of Pt working electrode to PPyNWA-PtNPs-SOx electrode, as illustrated in Scheme 1. AAO template was prepared by: (a) anodisation, (b) etching, (c) coating with gold, (d) electropolymerisation of pyrrole to form polypyrrole nanowire arrays (PPyNWA), (f) PtNPs decoration of PPyNWs, and (g) biofunctionalisation by adsorption of SOx.

#### 2.3.1. Fabrication of AAO Template

A two-step anodisation method was used with 0.2 mm thick (22 mm diameter) high-purity (99.99%) aluminum discs in oxalic acid (0.3 M) [20,21,22]. In the first step, an applied potential of 45 V was imposed for 4 h at 0 °C (Scheme 1a), and then the disk was placed in a mixture of 0.5% phosphoric acid and 0.5% chromic acid for 6 h at 60 °C to remove the oxide layer. In the second step, the anodisation was carried out for 6 h in oxalic acid (0.3 M), and the removal of the aluminum on the backside was accomplished by immersion in a 0.5 M copper chloride solution (Scheme 1b). The barrier layer removal and enlargement of the holes were achieved by placing the disk for 30 min in a 0.5 M phosphoric acid solution at 40 °C. The reverse side of the template was made conductive by vacuum-deposition of a gold layer (Scheme 1c). A thick layer of copper was deposited on the gold layer in a solution mixture which contained 0.5 M CuSO_4_ and 0.2 M H_3_BO_3_, with an applied potential of −2.0 V for 20 min [23].

#### 2.3.2. Growth of PPyNWA and Deposition of PtNPs

A polishing pad was used to polish the working electrode (Pt disk) with diamond slurries (1–15 µm, BASInc., Eatonville, WA, USA) to remove redox reaction products, followed by rinsing with Milli-Q water and subsequent sonication for 10 min in a mixed ethanol and water solution. Following vacuum-drying, the electrode was cleaned by cycling at a sweep rate of 75 mVs^−1^ for 10 min between −200 and +1450 mV versus Ag/AgCl in a 1.0 M H_2_SO_4_ solution [24]. The AAO template was cut into 4 mm diameter sections and fixed gently on the dried electrode with carbon paste (Scheme 1d). The PPyNWA were formed by electropolymerisation of pyrrole (various conc. 0.05–0.5 M) solution containing KCl (0.1 M) after removal of dissolved oxygen with nitrogen for 10 min at different applied current densities between 0.05 and 1.0 mA cm^−2^ and polymerisation periods ranging from 30 to 1200 s (Scheme 1d) [22]. The resulting polymer formed on the AAO template was reduced back for 120 s with an applied potential of −0.1 V in the pyrrole solution. After electropolymerisation, residual monomers were removed by rinsing the PPyNWA with Milli-Q water, and the AAO template was partly removed by placing it in a 0.5 M NaOH solution for 15 min, followed by subsequent washing with Milli-Q water (Scheme 1e). The PtNPs were deposited on the fresh PPyNWA by cycling the electrode at a scan rate of 50 mV s^−1^ between +0.20 and −0.20 V in a mixed H_2_SO_4_ (0.5 M) and K_2_PtCl_6_ (2.0 mM) solution (Scheme 1f) [25]. The use of 20 cycles provided optimum PtNP deposition.

#### 2.3.3. Adsorption of SOx

The adsorption of SOx on the resulting PPyNWA-PtNP electrode was achieved by immersion in 0–25 U/mL SOx solution in phosphate buffer (0.05 M) at 4 °C over a period of 1–24 h (Scheme 1g). Loosely attached SOx was removed by washing with Milli-Q water, and the electrode was stored at 4 °C in a phosphate buffer solution.

### 2.4. Amperometric Measurement with PPyNWA-PtNPs-SOx Biosensor

Cyclic voltammetry was performed within a potential range of −800 to +800 mV at a scan rate of 100 mV s^−1^ in a 0.05 M phosphate buffer solution (pH 7.0), with PPyNWA-PtNPs-SOx electrode as the working electrode, while the reference and auxiliary electrodes were as described in Section 2.2. The optimum amperometric response was obtained in 0.05 M phosphate buffer solution (pH 7.0) with the application of 700 mV, followed by repeated addition of an aliquot (20 µL) of 1 mM sulfite into a 2 mL buffer solution. All amperometric measurements were subsequently performed with an applied potential of 700 mV.

### 2.5. Optimisation of PPyNWA-PtNPs-SOx Biosensor

The fabrication of SOx-functionalised biosensor was optimised to establish the optimum working conditions. The influence of different formation and biofunctionalisation parameters was investigated, including pyrrole concentration for PPyNWA, chosen current density, polymerisation time, SOx concentration, PtNPs deposition, potential sweep range and sweep rate. Measurement conditions, including buffer concentration, pH, and temperature, were subsequently investigated.

### 2.6. Detection of Sulfite

The amperometric detection of sulfite was performed as described in Section 2.4. Beer and wine samples were analysed without any pretreatment, except that 0.05 M phosphate buffer was used to adjust pH to 7.0. For comparison, spectrophotometric measurements were also performed [26]. The coloured wine and beer samples were decolourised with activated charcoal. The charcoal was activated by placing it in a vacuum oven for 24 h and leaving it at room temperature for 30 min under vacuum prior to use. By using a sealed disposable syringe, an aliquot of each sample was treated by shaking vigorously with the added activated charcoal and by passing immediately through a 0.4 µm membrane connected to the syringe; a clear solution was obtained for spectrophotometric measurements.

Reagent SO_3_^−1^ K (1 level grey microspoon) was placed in a reaction cell. The cell was shaken vigorously until the reagent was completely dissolved. Pretreated beverage sample (3 mL) was added to the cell with a pipette, mixed, and left for 2 min before conducting photometric measurements.

## 3. Results and Discussion

### 3.1. Formation and Biofunctionalisation of NWA

The steps involved in the biofunctionalisation of the NWA with SOx are illustrated in Scheme 1, starting from the initial anodisation of aluminum for the formation of the AAO template to the SOx biofunctionalisation on the PtNP-decorated polypyrrole nanowire arrays (PtNPs-PPyNWA). Following formation of the AAO template, as described in Section 2.3.1 and in previous studies [22,27], vacuum-deposition was employed to deposit a uniform gold layer. Furthermore, the template was made more conductive and stronger by electrolytic deposition of a layer of copper [28], which also facilitated the subsequent attachment to the Pt working electrode with a carbon tape.

**Figure 1:**
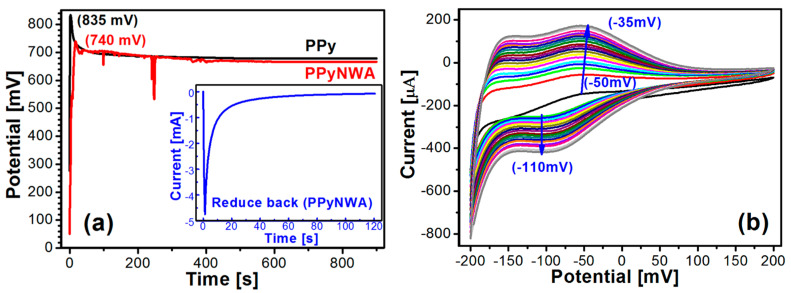
(**a**) Chronopotentiograms for growth of PPy film on a platinum electrode and of PPyNWA on an AAO template. Monomer solution contained 0.3 M Py and 0.2 M KCl; applied current density was 0.7 mA cm^−2^ for 900 s. Inset: PPyNWA reduced back with application of 0.1 V for 120 s. (**b**) Cyclic voltammograms of PtNP deposition on PPyNWA from +200 mV to −200 mV at scan rate of 50 mV s^−1^ for 20 cycles.

During the electropolymerisation, the pyrrole monomer was oxidised on the anode to form a polymer chain, which can be affected if oxygen is present in the solution [29]. Therefore, to avoid the effect of oxygen on the resulting PPy film, nitrogen was used to deoxygenate the monomer solution for 10 min prior to the electropolymerisation. The growth of PPy onto the gold- and copper-plated AAO template with the application of a current density of 0.7 mA cm^−2^ for 900 s is illustrated by the chronopotentiogram shown in Figure 1a. A rapid potential change from 50.2 to 742.8 mV was observed at the commencement of the electropolymerisation due to the formation of PPyNWA. Subsequent increase in film thickness [23] and an associated increase in PPy film conductivity lowered the stabilisation potential to 675.2 mV. The observed fluctuations were due to an irregular conductive channels for the flow of electrons resulting from the gold and copper layers on the AAO template, carbon tape, and the diffusion of pyrrole monomer inside the nano-holes during the electropolymerisation [22]. For comparison, the electropolymerisation of pyrrole on a bare Pt electrode was also performed with the same experimental conditions (Figure 1a). Evidently, the activation and stabilisation potentials obtained for PPyNWA formation were lower than those obtained for the PPy film, thus indicating that the PPyNWA was more conductive and easier to grow than the PPy film.

To achieve a more activated and uniformly packed structure, the PPyNWA was reduced back each time after its formation in the same solution by holding the electrode potential for 120 s at −0.1 V (Figure 1a inset). The increasing deposition of PtNPs onto the surface of PPyNWA is illustrated in Figure 1b by the increasing peak currents of the cyclic voltammograms with repeated cycles. It is obvious that the reduction and oxidation peak currents at −110 mV and −45 mV increased, respectively, with increasing number of cycles, indicating the deposition of well-dispersed PtNPs [22]. Notably, the peak potential for the reduction process at −110 mV remained reasonably stable, while the peak potential for the oxidation process shifted slightly from −50 mV to −35 mV as the number of cycles was increased due to an increase in deposited PtNPs thickness.

### 3.2. Morphological Study of PPyNWA-PtNPs by SEM

**Figure 2:**
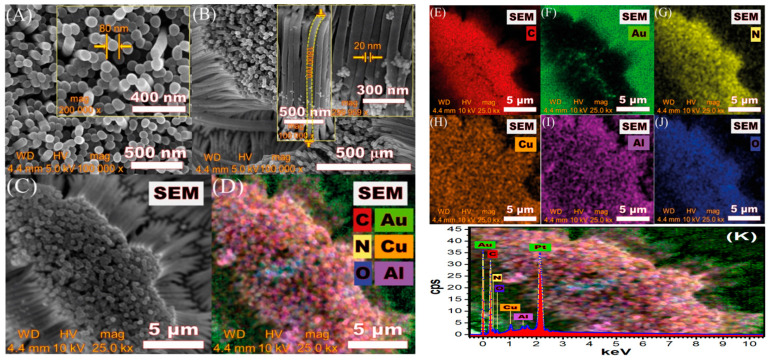
SEM image of (**A**) top view PPyNWA, inset: average diameter of PPyNWA at higher magnification; (**B**) overall view of PPyNWA-PtNPs, inset: cross-sectional view and average length of nanowires and diameter of PtNPs; (**C**) top view PPyNWA used for SEM mapping and EDX; (**D**–**J**) elemental distribution by SEM mapping; and (**K**) EDX spectrum of PPyNWA-PtNPs.

The images obtained for PPyNWA-PtNPs by scanning electron microscopy (SEM) are shown in Figure 2. It can be seen from the top view in Figure 2A that the nanowires were separated from each other and stood vertically to the gold substrate. The PPy nanowire had an average diameter of 80 nm, as revealed by the inset. Figure 2B reveals the overall morphology for the well-aligned PPyNWA and that the PtNPs were well-dispersed and partially embedded, with an approximate diameter estimated by SEM to be 18 nm (inset Figure 2B) [25].

The SEM and EDX mapping in Figure 2E–K revealed the elemental distribution in the PPyNWA-PtNPs. The presence of carbon (C) and nitrogen (N) due to the pyrrole ring in polypyrrole is obvious in Figure 2E,G, while the presence of gold (Au) and copper (Cu) [21] were evident in Figure 2F,H due to their use in the deposition layers to enhance the conductivity of PPyNWA. However, the presence of aluminum (Al) and oxygen (O) (Figure 2I,J) was possibly due to remnants of aluminum oxide left during the removal of the AAO template. Furthermore, the EDX spectrum in Figure 2K confirmed the presence of Pt due to the deposited PtNPs.

### 3.3. Electrochemical Impedance Analysis

**Figure 3:**
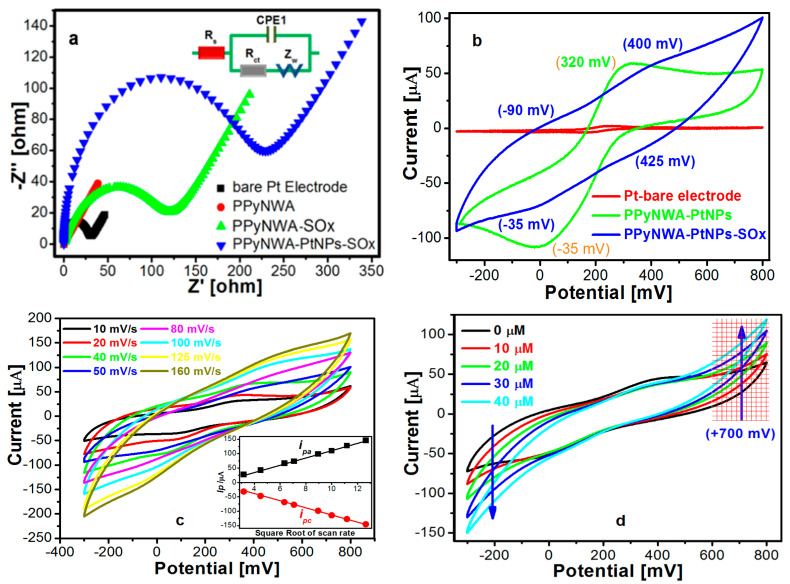
Electrochemical characterisation of PPyNWA-PtNPs-SOx biosensor. (**a**) Nyquist plots; (**b**) cyclic voltammograms at 50 mV s^−1^; (**c**) cyclic voltammograms at increasing scan rates, inset: *I_p_* vs. square root of scan rate, correlation coefficient (R^2^) was 0.9995; and (**d**) CVs of PPyNWA-PtNPs-SOx after addition of sulfite. Measurement solution: 0.05 M phosphate buffer in presence of 5 mM [Fe(CN)_6_]^3−/4−^.

The interfacial properties of the PPyNWA were investigated by conducting Nyquist impedance measurements in a 0.05M phosphate buffer solution (pH 7) which contained 5 mM [Fe(CN)_6_]^3−^/[Fe(CN)_6_]^4−^ as the electrochemical probe. In general, the electron-transfer kinetics are controlled by electron-transfer resistance, which is reflected by the dimension of the semicircle obtained at higher frequencies of the Nyquist plot. In contrast, the linear part at lower frequencies is indicative of the diffusion process of a redox probe at the electrode interface [30]. Figure 3a shows that the desired interfacial properties of the PPyNWA from the Nyquist plot were obtained by plotting the complex impedance (Z) versus frequency by using the Randle equivalent circuit. The charge transfer resistance (R_ct_) for the bare Pt electrode was 22.76 Ω, as calculated from the Nyquist plot. However, this value decreased dramatically to 0.00032 Ω for the PPyNWA and, thus, indicated that the transfer-of-electron process was accelerated in the presence of the redox probe. Biofunctionalisation of the PPyNWA with SOx increased the R_ct_ value to 117.93 Ω (small semi-circle) due to the lowering of the electrical conductivity, as expected by the presence of the enzyme. Even when the surface area of PPyNWA was improved with deposition of PtNPs to enable better entrapment of SOx, the R_ct_ increased further to 201.58 Ω (large semi-circle), which was reflective of the increased surface area of PPyNWA-PtNPs, thus resulting in a larger surface coverage with SOx molecules and an increased barrier effect on the electron-transfer kinetics [30].

### 3.4. Electrochemical Behaviour of PPyNWA-PtNPs-SOx

A comparison of the electrochemical behaviour of the PPyNWA-PtNPs electrode with and without biofunctionalised SOx was conducted in a phosphate buffer solution in the presence of the redox mediator. Figure 3b shows the CVs obtained with Pt, PPyNWA-PtNPs, and PPyNWA-PtNPs-SOx electrodes. The oxidation and reduction peaks observed at 320 mV and −35 mV, respectively, for the PPyNWA-PtNPs electrode were associated with the redox processes typically obtained with PPy. These redox peaks were shifted due to a change in surface chemistry, as the oxidation peak appeared at −100 mV instead of −35 mV, as reported previously by Ameer and Adeloju [26]. The Fe(CN)_6_^3−/4−^ redox process also contributed to the pronouncement of these peaks. The presence of SOx in the PPyNWA-PtNPs-SOx electrode was confirmed by the suppression of the previous two main peaks and the appearance of the oxidation and reduction peaks at 425 mV and −90 mV, respectively, while the other redox couple observed at 400 mV and −35 mV with the PPyNWA-PtNPs electrode was attributed to the redox processes of PPy with small shifts in potential.

Figure 3c demonstrates the involvement of surface-confined processes with the use of different scan rates at the PPyNWA-PtNPs-SOx electrode for the CV measurements. Highly symmetric and increasing redox peaks were obtained with increasing scan rates due to the reduction of the electroactive Fe(III) in the solution to Fe(II) in the forward scan, followed by full reoxidation to Fe(III) in the reverse scan [31]. A linear increase in the peak current with the square root of the scan rate was observed, as shown in Figure 3c, thus suggesting that diffusion-controlled reactions occurred at the PPyNWA-PtNPs-SOx electrode surface with the applied electrode potential [32].

In order to demonstrate the effectiveness of the biofunctionalisation of PPyNWA-PtNPs with SOx (as PPyNWA-PtNPs-SOx), its responsiveness to sulfite was investigated. In addition, the required applied potential for sulfite measurement was determined by adding different concentrations of the analyte. Evidently, the anodic peak currents increased with increasing sulfite concentrations, as shown in Figure 3d, while the other redox couple, which characterised the presence of SOx, remained unaffected, thus indicating that the biofunctionalisation of the PPyNWA-PtNPs with SOx did not hinder the electrode reactions and the associated transfer of electrons [32]. The observed increase in the reduction and oxidation peak currents were associated with the generation of hydrogen peroxide (H_2_O_2_), as given by the following reactions [22,26]:

(1) $${{{\mathrm{SO}}_{3}}^{2}}^{-}+O_{2}+H_{2}O\;\underset{\mathrm{SOx}}{\to }\;H_{2}O_{2}+{{{\mathrm{SO}}_{4}}^{2}}^{-}$$

$$H_{2}O_{2}+2H^{+}+2e^{-}\;\underset{\mathrm{electrode}}{\to }\;2H_{2}O\;(\mathrm{cathodic}\;\mathrm{current}\;\mathrm{response})$$

$$H_{2}O_{2}\;\underset{\mathrm{electrode}}{\to }\;2H^{+}+O_{2}+2e^{-}\;(\mathrm{anodic}\;\mathrm{current}\;\mathrm{response})$$

Consequently, the SOx present catalysed the generation of SO_4_^2−^ and H_2_O_2_ through the catalytic oxidation of the added sulfite. The highlighted area in the CVs (Figure 3d) shows that optimum response for amperometric measurement of sulfite could be obtained at a potential between +600 mV and +800 mV. Hence, the application of +700 mV was used for other sulfite measurements of the PPyNWA-PtNPs-SOx electrode.

### 3.5. Optimisation of the Nanobiosensor

#### 3.5.1. Deposition of PtNPs

**Figure 4:**
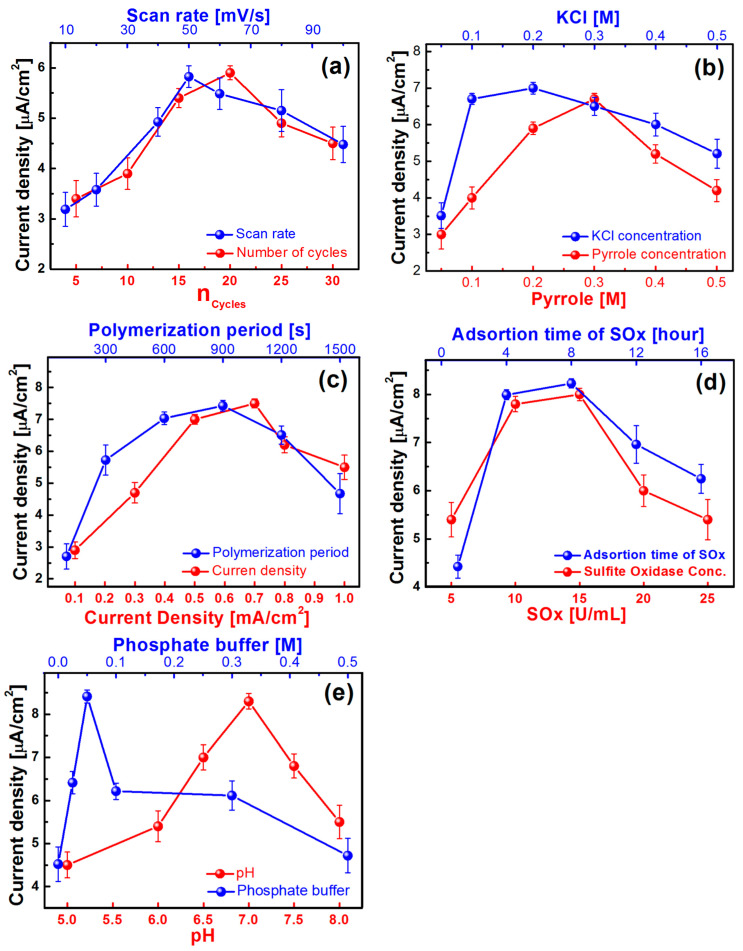
Consideration of factors influencing performance of the sulfite nanobiosensor. (**a**) PtNP deposition cycles and scan rate, (**b**) Py and KCl concentrations, (**c**) applied current density and polymerisation time, (**d**) sulfite oxidase concentration and adsorption time, and (**e**) pH and buffer concentration. Measurement with an applied potential of +700 mV; polymerisation conditions: 0.3 M Py, applied current density of 0.7 mA cm^−2^; PtNP deposition conditions: cycling at 50 mV s^−1^ for 20 cycles. Measurement solution contained 50 mM phosphate buffer and 10 µM sulfite.

The decoration of PPyNWA with PtNPs increased the available surface area for biofunctionalisation with SOx and acted as dispersed active centers. Both of these properties enhanced the oxidation of the generated hydrogen peroxide during the conversion of sulfite to sulfate [25]. Hence, the achievement of an adequate density of PtNPs is necessary to enable increased SOx loading and retention. The suitable density was attained by optimizing important factors, such as scan rate and PtNP deposition cycles. Figure 4a shows the influence of the number of the PtNP deposition cycles on the sulfite response. It can also be seen that the response continued to increase until the 20th cycle and decreased thereafter. The rapid decrease in the response after 20 cycles was due to the increased thickness of the nanoclusters, as well as the associated increase in the diffusion barrier, which limited the magnitude of the sulfite response [22]. It was, therefore, concluded that 20 cycles of PtNP deposition on the PPyNWs-PtNPs-SOx biosensor provided the optimum sulfite response. This condition was used for all subsequent investigations.

Furthermore, the chosen scan rate for the PtNP deposition was optimised. The influence of scan rate on the sulfite amperometric response is also illustrated in Figure 4a. Evidently, the response increased sharply with increasing scan rates from 20 to 50 mV s^−1^. However, beyond 50 mV s^−1^, the response decreased substantially due to increases in particle size and thickness. A scan rate of 50 mV s^−1^ was therefore chosen as the optimum rate for the deposition of PtNPs.

#### 3.5.2. Influence of Pyrrole and Potassium Chloride Concentration

The effective growth of the PPyNWA was dependent on the chosen Py concentration for the electropolymerisation. The influence of Py concentration on the growth of PPyNWA was investigated by examining its effect on the response obtained for sulfite with the PPyNWA-PtNPs-SOx biosensor. Evidently, as shown in Figure 4b, the amperometric response increased with the use of up to 0.3 M Py. At levels higher than this Py concentration, the PPy thickness increased, causing an increased diffusion barrier, resulting in the detection of less enzymatic products at the electrode and a decreased response. As the growth and thickness of the PPyNWA was highly dependent on the diffusion of Py into the AAO template pores [22], a lower Py concentration was unsuitable for effective PPyNWA growth, as confirmed by SEM. Consequently, 0.3 M Py was used for subsequent PPyNWA growth.

The growth of PPyNWA was also affected by the concentration of the electrolyte (KCl) used to facilitate the polymerisation of Py. The influence of the concentration of KCl in the monomer solution is shown in Figure 4b. Evidently, the increasing addition of KCl up to 0.2 M led to a sharp increase in the response, but it gradually decreased at a higher KCl concentration. A more rapid growth of PPy at the higher KCl concentration and the consequential increase in PPy thickness during the electropolymerisation was responsible for the decreased response [32]. Hence, 0.2 M KCl was used to optimise the PPyNWA growth.

#### 3.5.3. Applied Current Density and Polymerisation Time

The effect of applied current density used for PPyNWA growth on the sulfite response is illustrated in Figure 4c. The application of a current density of 0.7 mA cm^−2^ provided an optimum sulfite response. With the application of a lower current density, the PPyNWA formed was too short and weak, resulting in a less effective biofunctionalisation with SOx, in turn resulting in a low sulfite response. In contrast, the application of a current density higher than 0.7 mA cm^−2^ resulted in the growth of a much thicker PPyNWA and an associated increase in the diffusion barrier, which produced a lower sulfite response. The PPyNWA formed with an applied current density of 0.7 mA cm^−2^ produced an optimum sulfite response with the PPyNWA-PtNPs-SOx biosensor and provided the best reproducibility for the measurement. Consequently, the formation of PPyNWA-PtNPs-SOx biosensor with an applied current density of 0.7 mA cm^−2^ was chosen for further investigations.

The chosen polymerisation time also had significant influence on the PPyNWA formation and the corresponding diameter and length of the nanowires. Hence, the optimisation of the polymerisation time was necessary for achieving the appropriate diameter and length of the nanowires to ensure effective biofunctionalisation with SOx. The use of a polymerisation time up to 900 s produced an increasing sulfite response, as shown in Figure 4c. The sulfite response decreased with longer polymerisation times due to increased PPyNWA growth, even outside the AAO template. Consequently, this resulted in the growth of an excessive PPy layer on the top of the PPyNWA, as observed by SEM, and this rendered the removal of the template difficult. The resulting surface was also unsuitable for effective biofunctionalisation with SOx, resulting in a low sulfite response. The growth of the PPyNWA for all other investigations was, therefore, achieved with a polymerisation period of 900 s.

#### 3.5.4. SOx Concentration and Adsorption Period

The concentration of SOx used to biofunctionalise PPyNWA-PtNPs significantly influenced the sensitivity of the sulfite response. Figure 4d shows that the progressive increase of SOx concentration up to 10 U mL^−1^ demonstrated a corresponding increase in sulfite response. At concentrations greater than this, the increased enzyme loading and diffusion barrier resulted in a decreased sulfite response. The optimum concentration of 10 U mL^−1^ achieved in this study was higher than that reported in our previous studies [14,26], but this was due to the much larger surface area of the PPyNWA-PtNPs and the associated increase in SOx loading. For this reason, 10 U mL^−1^ of SOx was used to biofunctionalise the PPyNWA-PtNPs for further investigations.

The performance of the PPyNWA-PtNPs-SOx biosensor was significantly influenced by the SOx molecule adsorbed onto the PPyNWA-PtNPs, which is dependent on the chosen adsorption time. Figure 4d shows that an adsorption time of 8 h provided the optimum sulfite response, and the use of longer adsorption times resulted in a decrease in the response. This was due to excessive SOx loading, which blocked the accessibility of the enzymatic product to the surface of the PPyNWA-PtNPs.

#### 3.5.5. Influence of pH and Buffer Concentration

The use of the optimised PPyNWA-PtNPs-SOx biosensor for sulfite measurement was significantly dependent on the solution pH, as the SOx activity was critically dependent on the pH. The best solution pH, as shown in Figure 4e, was between 6.5 and 7.5, but the optimum response was obtained at pH 7.0, which agreed with a previously reported study [26]. Protonation (low pH) and deprotonation (high pH) at the active sites of SOx accounted for the decreased sulfite response at lower and higher solution pH levels. A change in the conformational geometry and ionic interactions at the active SOx sites may have also contributed to the observed reduction. In addition, above pH 7.0, the degradation of the polymer backbone by OH^─^ and the reduction of PPy conductivity may have been contributing factors.

Figure 4e also illustrates that the chosen phosphate buffer concentration can significantly influence the sensitivity of the sulfite response obtained with the PPyNWA-PtNPs-SOx biosensor. Notably, an increase in buffer concentration beyond 0.05 M resulted in a decrease in the sulfite response, which was attributed to the presence of excessive bulky ions in the solution [32]. Therefore, a low buffer concentration (0.05 M) was used to obtain optimum sensitivity for the sulfite response. However, if required, a buffer concentration up to 0.3 M can be used if a small decrease in sensitivity is not of concern.

### 3.6. Analytical Performance and Applications

**Figure 5:**
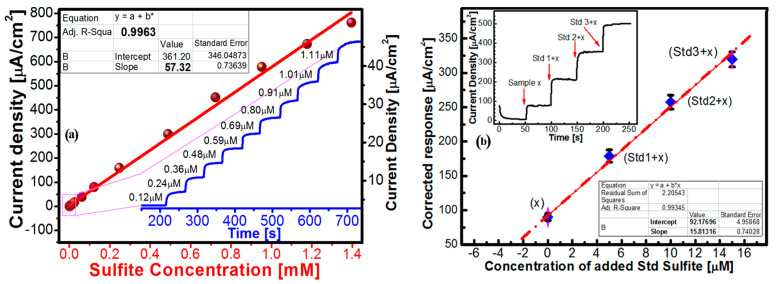
(**a**) Typical linear range achieved for sulfite response; inset shows the successive amperometric response of the PPyNWA-PtNPs-SOx biosensor to sulfite. (**b**) Amperometric sulfite determination in wine (W1) with PPyNWA-PtNPs-SOx biosensor; inset shows the typical chronoamperograms for sulfite quantification in a wine sample by standard additions method.

SOx-based amperometric biosensors measure sulfite concentrations in samples by detection of the H_2_O_2_ generated from the enzyme-catalysed reaction, as previously presented in Equation (1). In this study, the oxidation of H_2_O_2_ generated was monitored for the measurement and quantification of sulfite. After optimisation, the PPyNWA-PtNPs-SOx biosensor was used to measure the increasing concentration of sulfite to verify its suitability for determination in real samples. Figure 5a (inset) shows that the successive injections of sulfite effected a stepwise increase in the response, with a rapid response time of 2s, thus demonstrating that the biofunctionalised PPyNWA-PtNPs-SOx biosensor had an excellent electrocatalytic behaviour. The calibration plot in Figure 5a also shows that the response increased with increasing sulfite concentrations between 0.12 and 1200 μM. The sensitivity within this linear range was 57.33 μA cm^−2^ mM^−1^, and the correlation coefficient (R^2^) was 0.99638. The limit of detection was 12.35 nM. The limit of detection and linear range achieved in this study were much lower and wider, respectively, than those reported for other SOx-based sulfite nanobiosensors [33].

The application of the PPyNWA-PtNPs-SOx biosensor for sulfite determination in beer and wine samples was conducted to verify its practical use for real samples. A standard additions method was employed, which was based on the injection of known sulfite concentrations into the samples within the established linear range of the biosensor. Figure 5b shows the standard addition plot with extrapolation used to determine sulfite concentration in wine (W1). Furthermore, typical amperometric responses obtained for a wine sample addition into a buffer solution, followed by standard additions of sulfite, showed subsequent increases in the response (inset Figure 5b). As demonstrated by the data in Table 1, a relatively good agreement was obtained for the sulfite results obtained with the biosensor and the standard photometric method. However, considerable differences were observed in coloured samples particularly red wine, possibly due to the effect of the coloured samples on the photometric measurements. The removal of the sample colour by decolourisation with charcoal may have lowered the sulfite concentration in the sample. In contrast, no apparent interference was observed for the amperometric measurement due to the specificity and selectivity induced through the biofunctionalisation with SOx. Obviously, the PPyNWA-PtNPs-SOx biosensor achieved superior performance in terms of sensitivity, detection limit, and fast response time, as well as its ability to handle coloured or turbid samples. In addition, a recovery study was conducted by spiking the wine and beer samples with known sulfite concentrations, and a recovery efficiency of 97–103% was achieved with the nanobiosensor.

### 3.7. Interferences, Reproducibility, and Stability

**Figure 6:**
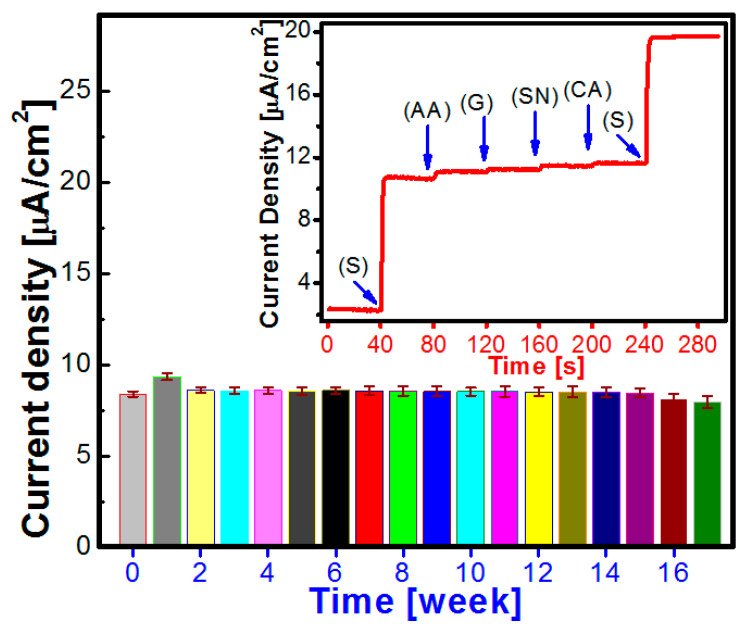
Influence of interferants (inset) and storage time on nanobiosensor response for sulfite. Measurement at +700 mV (*n* = 3); polymerisation with 0.3 M Py for 900s and 0.7 mA cm^−2^; PtNPs deposition for 20 cycles at a scan rate of 50 mV s^−1^. Measurement solution was 0.05 M phosphate buffer (pH 7.0) and 10 µM sulfite.

The commonly present electroactive species in food and beverages, such as ascorbic acid (AA), glucose (G), sodium nitrate (SN), and citric acid (CA), can interfere with the reliable determination of sulfite. For this reason, we investigated the effects of these substances on sulfite measurements with the PPyNWA-PtNPs-SOx biosensor. The measurement of 10 µM sulfite was used for the investigation, followed by successive injections of 10 µM of AA, G, SN, and CA, and then followed by a repeat addition of 10 µM sulfite, as shown in Figure 6 (inset). It was also obvious that the addition of each of the interferants resulted in small increases in the sulfite response. The relative increase in sulfite response caused by the interferants was between 1.8 and 4.5%, thus demonstrating that the PPyNWA-PtNPs-SOx biosensor exhibited a high level of selectivity. The observed level of interference had a minimal effect on the measurement of the sulfite, as demonstrated by the second addition of sulfite (S). In addition, the use of the standard additions approach for quantification of the sulfite in the samples should compensate for this and other matrix effects in the sample.

The PPyNWA-PtNPs-SOx sulfite biosensor was evaluated for short- and long-term stability by repeated use and storage over a period of 18 weeks (4.5 months). In one approach, the stability of the biosensor was evaluated by recording the cyclic voltammogram of sulfite in the presence of [Fe(CN)_6_]^3−/4−^ at 3 min intervals. The anodic peak current obtained for sulfite in the CV was reasonably constant with repeated measurements (2.5% rsd). On the other hand, the long-term stability of the biosensor was evaluated by storing it at 4 °C in a phosphate buffer solution (0.05 M) and using it weekly for sulfite measurements. After the first week, as shown in Figure 6, the sulfite response increased, possibly as a consequence of an increase in the porosity of the PPy, but it remained reasonably stable between weeks 2 and 15. There was, on average, only a 4% decrease in sulfite response during this period. However, at weeks 16 and 17, the response decreased slightly as a consequence of some SOx leakage from the nanobiosensor, resulting in a lower sulfite response. Nevertheless, the biosensor can still be used at this stage, provided a standard additions method is used for quantification of the sulfite concentration.

Three nanobiosensors prepared by the same procedure were used to evaluate the reproducibility of the sulfite measurements. The measurement of sulfite response with each nanobiosensor was repeated nine separate times. The sulfite responses obtained with the nine repeat measurements for the three individual biosensors were very reproducible, with a 2.4% rsd (*n* = 9), indicating that sulfite measurement with the PPyNWA-PtNPs-SOx nanobiosensors is highly reproducible.

Table 2 summarises the differences in the preparation, measurement conditions, and performances of the PPyNWA-PtNPs-SOx nanobiosensor with those of other PPy-based sulfite biosensors. Evidently, the PPyNWA-PtNPs-SOx nanobiosensor is not only novel on the basis of its process of fabrication and measurement, but it is far more superior in terms of its response time, linear concentration range, minimum detectable concentration, and stability. The growth of PtNPs on top of the PPyNWA and the subsequent long-term adsorption (8 hrs) of SOx on the PtNPs was responsible for the much-improved stability of the biosensors. Compared with our earlier study [14], where the PPy-SOx biosensor was stable for only 4 hrs, the improvement in stability achieved with the PPyNWA-PtNPs-SOx nanobiosensor in this study represents a major achievement for sulfite detection.

## 4. Conclusions

We have successfully demonstrated in this study that the biofunctionalisation of a polypyrrole nanowire array (PPyNWA) with SOx aided by decoration with platinum nanoparticles (PtNPs) is very effective for achieving ultrasensitive amperometric detection of sulfite. The presence of the PtNPs provided improved surface area and dispersed active centres that enabled much higher SOx loading (10 U mL^−1^) and resulted in a substantial improvement in the amperometric detection of sulfite. The PPyNWA-PtNPs-SOx nanobiosensor achieved excellent analytical performance, as demonstrated by a relatively fast response time of 2 s, a limit of detection of 12.35 nM, and a linear concentration range between 0.12 and 1200 μM, with a corresponding sensitivity of 57.33 μA cm^−2^ mM^−1^. Furthermore, with continuous use, the response of the nanobiosensor was relatively reproducible up to 24 h and could be stored for repeated use at 4 °C in 0.05 M phosphate buffer for 18 weeks. The performance of the nanobiosensor was not affected by common interfering substances, such as ascorbic acid, glucose, citric acid, or sodium nitrate. The nanobiosensor was also successfully applied to sulfite determination in alcoholic beverages (beer and wine), and a good agreement was achieved for the sulfite results obtained for beer and wine samples by both the nanobiosensor and spectrophotometric method. However, for red wine and beer, the nanobiosensor provided more reliable results than the spectrophotometric method, which was seriously affected by the colour of the samples.

## Figures and Tables

**Table 1:** Concentrations of sulfite obtained in beer and wine samples with the biosensor and the photometric method.

Sample Code	Sample	Alcohol Content (% Alc/Vol)	Photometric Results (µM), n = 3	Biosensor Results (µM), n = 3
BE1	Beer	3.6	21.25 ± 0.70	21.95 ± 0.65
BE2	Beer	5.4	17.50 ± 0.52	17.82 ± 0.60
BE3	Beer	3.5	16.25 ± 0.65	16.47 ± 0.73
WI1	Yellow wine	11.5 (contains preservative 220)	43.75 ±0.40	44.11 ± 0.30
WI2	White wine	14.5	6.25 ± 0.46	6.40 ± 0.52

**Table 2:** Performances of PPy-based sulfite biosensors prepared under different conditions.

Parameter/Condition	This Work	PPy Sulfite Nanobiosensor [16]	PPy Biosensor [26]	PPy Flow Biosensor [15]
Electrode				
Working electrode material	Nanowires array	Pt—No nanowires or array	Pt—No nanowires or array	Pt—No nanowires or arrays
Immobilisation of SOx				
Mode of immobilisation	Adsorption for 8 h on top of PtNPs grown on top of PPyNWA (SOx adsorbed outside)	Entrapment within ultrathin PPy film grown on top of PtNPs (SOx trapped inside)	Entrapment within ultrathin PPy film grown on Pt electrode (SOx trapped inside)	Entrapment within PPy film grown on Pt electrode (SOx trapped inside)
SOx concentration	10 U mL^−1^	5 U mL^−1^	5 U mL^−1^	10 U mL^−1^
Location of PtNPs	On top of PPyNWA	Under PPy film	No PtNPs	No PtNPs
Location of SOx	On top of PtNPs	Inside PPy film	Inside PPy film	Inside PPy film
PPy formation conditions				
Polymerisation time	900 s	60 s	120 s	300 s
Py concentration	0.3 M	0.2 M	0.1 M	0.5 M
Applied current density	0.7 mA cm^−2^	0.3 mA cm^−2^	0.2 mA cm^−2^	0.5 mA cm^−2^
PPy formation	On NWA (without PtNPs)	On top of PtNPs	On top of Pt disc electrode	On top of Pt disc electrode
Measurement conditions				
Phosphate buffer solution	50 mM	25 mM	50 mM and 10 mM KCl	100 mM and 500 mM KCl
Applied potential	+700 mV	None	−700 mV	−900 mV
Detection mode	Amperometry	Potentiometry	Amperometry	Flow Amperometry
Performance				
Response time	2 s	3–5 s	5–7 s	Flow-rate-dependent
Linear concentration range	0.12 to 1200 μM	0.75 to 65 μM	0.9 to 400 μM	0 to 200 mg/L
Minimum detectable concentration	0.12 μM	0.75 μM	0.9 μM	N/A
Stability	18 weeks	12–13 weeks	30 days	21 days
Number of fabrication steps	7	3	1	1

## Data Availability

Available on request from authors.
